# Enhanced disgust generalization in obsessive–compulsive disorder is related to insula and putamen hyperactivity

**DOI:** 10.1017/S0033291725000728

**Published:** 2025-04-14

**Authors:** Juntong Liu, Jinxia Wang, Yuchen Song, Benjamin Becker, Xianchao Ming, Yi Lei

**Affiliations:** 1 College of Psychology, Shenzhen University, Shenzhen, China; 2 Institution for Brain and Psychological Science, Sichuan Normal University, Chengdu, China; 3 Center for Neurogenetics, Shenzhen Institute of Neuroscience, Shenzhen, China; 4 Faculty of Education and Psychology, University of Jyväskylä, Jyväskylä, Finland; 5 State Key Laboratory of Brain and Cognitive Sciences, Department of Psychology, The University of Hong Kong, Hong Kong, China; 6 School of Psychology, South China Normal University, Guangzhou, China

**Keywords:** contamination concern, disgust, acquisition, generalization, insula, obsessive-complusive disorder, putamen, fMRI, ROI, contamination-related fear, disgust learning, conditioned responses

## Abstract

**Background:**

Compulsive cleaning is a characteristic symptom of a particular subtype of obsessive–compulsive disorder (OCD) and is often accompanied by intense disgust. While overgeneralization of threat is a key factor in the development of obsessive–compulsive symptoms, previous studies have primarily focused on fear generalization and have rarely examined disgust generalization. A systematic determination of the behavioral and neural mechanisms underlying disgust generalization in individuals with contamination concern is crucial for enhancing our understanding of OCD.

**Method:**

In this study, we recruited 27 individuals with high contamination concerns and 30 individuals with low contamination concerns. Both groups performed a disgust generalization task while undergoing functional magnetic resonance imaging (fMRI).

**Results:**

The results revealed that individuals with high contamination concern had higher disgust expectancy scores for the generalization stimulus GS4 (the stimulus most similar to CS+) and exhibited higher levels of activation in the left insula and left putamen. Moreover, the activation of the left insula and putamen were positively correlated with a questionnaire core of the ratings of disgust and also positively correlated with the expectancy rating of CS+ during the generalization stage.

**Conclusion:**

Hyperactivation of the insula and putamen during disgust generalization neutrally mediates the higher degree of disgust generalization in subclinical OCD individuals. This study indicates that altered disgust generalization plays an important role in individuals with high contamination concerns and provides evidence of the neural mechanisms involved. These insights may serve as a basis for further exploration of the pathogenesis of OCD in the future.

## Introduction

Obsessive–compulsive disorder (OCD) is a disabling disorder characterized by intrusive obsessive thinking or/and repetitive compulsive behaviors (Diagnostic and statistical manual of mental disorders, 1994). It is estimated that around 2% of the world’s population suffers from OCD and that almost all affected individuals experience intrusive thoughts and strongly aversive emotional states and have a strongly increased risk for comorbid mental and physical disorders (Adam, Meinlschmidt, Gloster, & Lieb, 2012; Murphy et al., 2010; Ruscio, Stein, Chiu, & Kessler, 2010). Patients with OCD present with various symptoms and behaviors such as contamination obsessions with washing/cleaning compulsions and doubting obsessions with checking compulsions (Matsunaga et al., 2008). The boundaries between these OCD subtypes are not always clear, and many patients exhibit obsessions and compulsions from different subtypes (Starcevic & Brakoulias, 2008). However, contamination-related fears are one of the most common and prominent symptoms of OCD, affecting up to 50% of individuals with the disorder (referred to as contamination-based OCD or C-OCD) (Markarian et al., 2010). Patients with this subtype experience an intense, irrational fear of germs, dirt, toxic substances or even mental contamination, which drives them to engage in excessive cleaning rituals or avoidance behaviors (Rachman, 2004).

Disgust is an emotional response to stimuli with contaminating properties, serving to protect an individual’s health from harmful substances (Mason & Richardson, 2010). In patients with compulsive cleaning behaviors, the onset of symptoms is often accompanied by a strong disgust for contamination (Bhikram, Abi-Jaoude, & Sandor, 2017). Consequently, disgust is considered a highly relevant emotion in obsessive–compulsive symptoms and may play a significant role in the emotional processing associated with the compulsive cleaning subtype (Armstrong & Olatunji, 2017; Broderick, Grisham, & Weidemann, 2013; Cisler, Brady, Olatunji, & Lohr, 2010; Ludvik, Boschen, & Neumann, 2015; Olatunji & Kim, 2024). Given the co-occurrence of anxiety and OCD symptoms, as well as the hypothesis of shared etiopathological factors, exploring the relationship between dysregulations in Pavlovian fear conditioning and OCD symptoms is crucial for a deeper understanding of the disorder (Armstrong, McClenahan, Kittle, & Olatunji, 2014). However, research on generalization remains limited. Rouhani et al. (2019) demonstrated that individuals with OCD exhibit impaired reward generalization but intact punishment generalization, suggesting domain-specific deficits in generalization rather than a broad impairment. This specificity warrants further investigation into the mechanisms underlying atypical generalization patterns in OCD. Previous studies have not observed excessive generalization in individuals with high compulsion which may be due to the fact that fear of physical harm is less relevant to OCD (Kaczkurkin & Lissek, 2013). Additionally, disgust learning may play a key role in contamination-based OCD. The exaggerated perceptions of contamination in many OCD patients may stem from abnormal disgust learning processes (Armstrong & Olatunji, 2017). Therefore, exploring the characteristics of disgust generalization in individuals with obsessive–compulsive cleaning is crucial for identifying its etiologic factors and warrants further investigation.

Pavlovian conditioning theory is a classic framework for studying generalization. Meanwhile, Pavlov’s disgust-conditioned reflex model has been successfully applied to determine the pathological mechanisms related to disgust in pollution-based OCD (Stein, Liu, Shapira, & Goodman, 2001). In the laboratory, researchers repeatedly pair conditioned stimuli (CS) (For example, a sound of 300 Hz as CS+, a sound of 800 Hz as CS-) with unconditioned stimuli (US) that elicit disgust responses. When a disgust response occurs in response to the CS alone, it indicates that the individual has successfully completed the acquisition (Herry & Johansen, 2014). CS paired with the US are referred to as CS+, while those not paired with the US are termed as CS−. Generalization is said to occur when the individual also exhibits responses to the GS (Lissek et al., 2014). In generalization studies, after an individual has completed acquisition, they are presented with a series of generalized stimuli (GS,400 Hz, 500 Hz tone) that are similar to CS+. GSs are used to determine whether conditioned responses to a primary CS+ extend to similar stimuli. Assessing generalization is crucial for understanding how individuals apply learned responses to new but comparable contexts. These insights are essential for clarifying and managing psychological disorders involving conditioned reflexes, such as anxiety disorders and OCD. For instance, after experiencing food poisoning from spoiled seafood, a person may develop disgust toward foods with a similar smell or appearance – an adaptive response to minimize health risks. However, this disgust can irrationally generalize to all types of food and eating situations, leading to the avoidance of many safe foods and restaurants due to perceived danger. The closer a GS (For example, GS4, 400 Hz tone) is to the CS+ (a sound of 300 Hz) in terms of perceptual features, the more likely it is to elicit a similar disgust response. Because GS4 is very similar to the CS+, the conditioned response to GS4 would be high but slightly less than the response to the CS+. Whereas, GS1 (a lower similarity to CS+, 700 Hz tone) is considerably different from the CS+. As a result, GS1 elicits a much weaker fear response than GS4 because it is less similar to the CS+.

The neural mechanisms associated with disgust in healthy subjects and individuals with obsessive–compulsive tendencies have been explored in various studies. MRI studies have demonstrated that the experience of disgust is encoded in widespread brain systems, with the insula, amygdala, striatal and prefrontal regions being commonly identified (Gan et al., 2022). Additionally, in comparison to control subjects, the bilateral insula in OCD patients exhibited an increased response to disgusting images control subjects, a difference not observed with threatening images (Schienle et al., 2006). Further findings suggest that the anterior insula plays a central role in modulating the contaminating obsessive–compulsive inhibition of responses to disgusting images (Berlin et al., 2015).

To date, studies on the neural basis of disgust generalization in individuals with OCD are scarce. Investigating the characteristics and neural basis of disgust generalization in individuals with OCD will not only enrich our understanding of the disorder but also provide new targets for clinical intervention. However, the heterogeneity of OCD symptoms and the complexity of the disorder’s etiology present several limitations when studying clinical samples. These limitations include variations in medication duration, comorbidities and other confounding factors. Therefore, utilizing nonclinical samples, specifically individuals with high yet subclinical levels of obsessive–compulsive tendencies, can facilitate more focused research on the underlying mechanisms of the disorders and subsequently guide the determination of novel treatment approaches(Kou et al., 2022; Wong & Beckers, 2021; Xin et al., 2020).

In this study, we aim to examine the characteristics and neural basis of disgust generalization in individuals with high and low compulsive contamination concerns. To this end, we recruited participants with high and low compulsive contamination concerns. Both groups subsequently underwent Pavlovian disgust acquisition and disgust generalization with concomitant fMRI acquisition. Previous studies have shown that individuals with high compulsive contamination concerns often exhibit high levels of disgust sensitivity (Gurak Thayer et al., 2021; Rickelt et al., 2019). Meanwhile, the level of activation of the insula is closely related to disgust and closely associated with OCD symptoms (Li et al., 2021). Consequently, we hypothesized that (1) compared to the low contamination concern group, the high compulsive contamination concern group would report a higher expectancy rating towards generalized stimuli and experience a higher level of disgust and that (2) the activation level of brain regions related to disgust (such as insula) would be higher in the group with high contamination concerns.

## Method and materials

### Participants

300 copies of the Padua Inventory-Contamination of obsessive–compulsive Disorder Subscale (PI-COWC) (Burns, Keortge, Formea, & Sternberger, 1996) were distributed through campus advertising to recruit student participants with high and low contamination concerns, based on a previous study (Armstrong & Olatunji, 2017). The inclusion criteria for the high tendency group was a PI-COWC score greater than 13, while the low tendency group required a PI-COWC score less than 7. All participants had normal or corrected vision and had no history of psychiatric or neurological diseases (according to self-report). The above recruitment methods are consistent with the research of Wang et al. (2024), but the samples do not overlap (J. Wang et al., 2024). We used G-power 3.1 to estimate power for behavioral analyses using repeated measures, within-between interaction ANOVAs (two groups and six measurements) with an effect size of Cohen’s f = 0.25 (Kang, 2015; Y. Wang et al., 2024) and α = 0.05. A total of 28 participants were required based on imaging measures. After the initial examination, three subjects were excluded for failing to acquire which type of CS would be followed by disgust stimuli (Armstrong & Olatunji, 2017), and one subject was excluded due to excessive head movement during the generalization phase. Therefore, a total of 57 subjects were included in the final analysis. Among them, there were 27 individuals (20.41 ± 1.45, female = 24) in the high-compulsive contamination concern group and 30 individuals (19.80 ± 1.16, female = 23) in the low-compulsive contamination concern group. Participants provided informed consent before the study and received compensation upon completion. The study was approved by the Ethics Committee of Sichuan Normal University.

### Experimental design

The study was divided into two phases: the acquisition phase and the generalization phase. The acquisition phase included two stimulus categories (CS+ and CS−), while the generalization phase included six stimulus categories (CS−, GS1, GS2, GS3, GS4 and CS+).

### Experimental materials

Circles of different sizes were used as experimental materials (Lissek et al., 2008). Specifically, ten circles with diameters increasing in 20% increments from 5.08 cm to 14.22 cm were used, with either the largest or smallest circle serving as the CS+. The assignment of the CS+ was counterbalanced between subjects. See Supplementary Figure S1. for the sample stimulus.

See more descriptions about the US in supplementary materials.

### Scales

Each subject completed the following self-report scale (see more information in supplementary materials):

1. **Padua Inventory Contamination of obsessive**–**compulsive Disorder Subscale (PI-COWC):** The Padua Inventory is a self-report measure of obsessive–compulsive and compulsive symptoms (Burns, Keortge, Formea, & Sternberger, 1996), where each question assesses the degree to which obsessive–compulsive symptoms interfere with the individual’s life, using a 5-point scale ranging from 0 (absent) to 4 (severe). assessment. The present study used its pollution OCD subscale of 10 questions for the degree of obsessive compulsiveness in terms of pollution (in the current sample: Cronbach’s ɑ = 0.94).
2. **The Revised obsessive**–**compulsive Inventory (OCI-R):** The revised OCI-R measures the main symptoms of OCD on six dimensions: checking, cleaning, ordering, hoarding, obsessing and neutralizing (Foa et al., 2002), with each question assessed in terms of frequency of occurrence and degree of distress, with the frequency of occurrence using a 5-point scale of 0 (never) − 4 (almost always) on a 5-point scale (in the current sample: Cronbach’s ɑ = 0.91), and the level of distress was assessed using a 5-point scale of 0 (not at all distressing) − 4 (extremely distressing) (in the current sample: Cronbach’s ɑ = 0.94).
3. **State–Trait Anxiety Inventory (STAI):** The State–Trait Anxiety Inventory (STAI) consists of two 20-item self-report scales (Sydeman, 2018). The SAI is used to measure the intensity of anxiety at a particular moment in time, and the TAI asks people to describe their overall feelings based on how often they experience specific anxiety symptoms (in the current sample: Cronbach’s ɑ = 0.97).
4. **Disgust Sensitivity Scale (DS-R):** The Disgust Sensitivity Scale was used to assess the sensitivity of subjects’ responses to disgusting stimuli (Olatunji et al., 2007). The scale categorizes disgust into three dimensions: core disgust, animal remainder and interpersonal contamination-based disgust and consists of 27 items, two of which are lie-detector questions, and all items were rated on a 0–4 scale, with higher ratings representing greater sensitivity (in the current sample: Cronbach’s ɑ = 0.89).

### Experimental procedure

The formal experiment consisted of three phases: the habituation phase, the disgust acquisition phase and the disgust generalization phase performing magnetic resonance scanning in all three stages. The three stages are carried out continuously.

During the habituation phase, the CS+, CS− and each of the four GSs were presented 20 times in a pseudo-randomized order, ensuring that no stimulus appeared consecutively more than twice (Y. Wang et al., 2024). This resulted in a total of 120 trials during this stage. During each time, CS was presented for 4 seconds and followed by a fixation for 2.4–4.8 seconds as inter-trail intervals. Subjects were only required to passively view and familiarize themselves with each graphic. The entire stage takes about 15 minutes.

**Figure 1.**
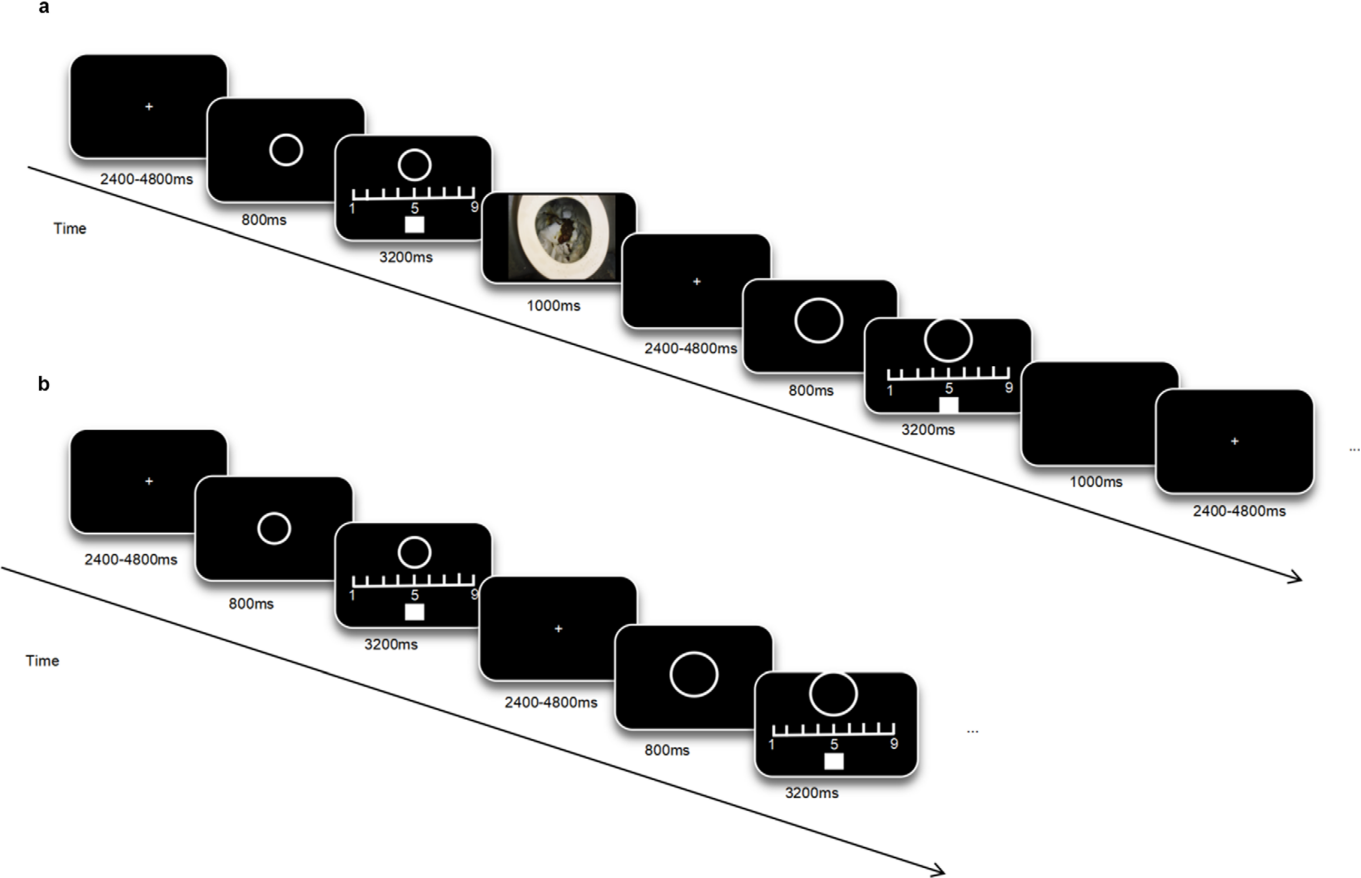
**Experimental procedure diagram of acquisition phase and generalization phase**. (a) In acquisition phase, subjects were required to score the likelihood of a disgusting stimulus following different stimuli. (b) In the generalization phase only a small probability of following the aversive pictures after CS+, subjects need to rate the generalized stimuli according to the laws learned in the acquisition phase.

In the acquisition phase, a randomly selected fixation point was displayed for 2.4–4.8 seconds, followed by the presentation of a CS alone for 800 ms. Participants were then instructed to provide their ratings within 3200 ms, during which the CS remained on the screen. In total, each CS was presented for 4000 ms. The CS was presented in a pseudorandomized order so that no more than two of the same CS appeared consecutively. Subjects were required to rate the likelihood of a disgusting picture following CS on a scale of 1 to 9. Participants adjusted the position of a square on the screen by pressing a button, with the square’s final position reflecting their expected level of unconditional stimulation. A disgusting picture (US) appeared and lasted for 1 second after the CS+, while no disgusting picture followed the CS−. Subsequently, a fixation point appears for 2.4–4.8 seconds (ITI). Each type of CS was presented 15 times, with an 80% reinforcement rate for CS+, resulting in a total of 30 trials. At the end of the phase, subjects rated the two types of CS on a disgusting scale of 1 to 9, see Figure 1a. After the end of this phase, the researcher informed the subject to take a short break and then entered the generalization phase. The entire stage takes about 5 minutes.

The generalization phase was similar to the acquisition phase. Random fixation points were first presented for 2.4–4.8 seconds, followed by the stimulus. Subjects rated the likelihood that the stimulus would be followed by a disgusting picture. During this phase, CS+ appeared 15 times, accompanied by the US five times to prevent premature extinction of disgust to CS+ (Wang, Sun, Becker, & Lei, 2022). The CS− and each GS were presented 10 times, resulting in a total of 65 trials. The order of stimulus presentation was pseudo-randomized, with no more than two consecutive occurrences of the same type of stimulus. No rest during the entire generalization process. Consistent with the acquisition phase, subjects rated the two types of CS and each GS retrospectively on a disgusting scale after the end of the generalization phase, see Figure 1b. The entire stage takes about 10 minutes.

### fMRI data acquisition

The fMRI data for this experiment were acquired using a Siemens Prisma 3.0 T MRI scanner (Siemens, Erlangen, Germany) (see more information in supplementary materials).

## Data analysis

### Analysis of behavioral data

A 2 (group: high/low obsessive–compulsive tendency) × 2 (stimulus: CS+/CS−) repeated-measures ANOVA was conducted to analyze the expected ratings of subjects during the acquisition phase. Similarly, a 2 (group: high/low obsessive–compulsive tendency) × 2 (stimulus: CS+/CS−) repeated-measures ANOVA was performed on the disgust ratings for the different CSs during the acquisition phase.

To examine changes in ratings across the time course of the generalization phase, each trial was included in the analysis. We performed linear mixed models (LMM, “lme4”) in R v.4.1.1 (R Development Core Team, 2012) for the behavioral analyses on prediction ratings during the generalization stage. To account for individual differences and variations across multiple levels of each variable, a 2 (group: high/low contamination concern) × 6 (stimuli: CS-/GS1/GS2/GS3/GS4/CS+) × 10 (trials: 1/2/3/4/5/6/7/8/9/10) linear mixed model analysis was conducted. Finally, a 2 (group: high/low contamination concern) × 6 (stimuli: CS-/GS1/GS2/GS3/GS4/CS+) repeated-measures ANOVA was performed on the disgusting scores during the generalization phase. For all analyses, a significance level of p < 0.05 was defined. In case of violation of sphericity, Greenhouse–Geisser epsilon (GG-ε) and uncorrected degrees of freedom are reported (Ahrens et al., 2016).

### Analysis of fMRI data

#### fMRI data preprocessing

fMRI data preprocessing was conducted using MATLAB version 2021b (The MathWorks Inc., Natick, USA) and DPABI (RRID), an open-source software package derived from DPARSF and REST (source code available at http://rfmri.org/DPABI). See more information in supplementary materials.

#### First-level and second-level analysis

**First-Level Analysis**: After completing preprocessing, first-level analyses were conducted using the SPM12 toolbox in MATLAB2019a (Wellcome Department of Imaging Neuroscience, London; http://www.fl.ion.ucl.ac.uk/spm). A general linear model (GLM) was applied to create contrasts for each CSs and GSs during the generalization phase (with CS+ in the generalization phase excluding trials accompanied by US). MATLAB-based code was used to batch-process these analyses for efficiency across subjects.

**Second-Level Analyses**: Using the CON files generated from the first-level analyses, second-level analyses were performed with the SPM12 toolkit. For the generalization phase, a 2 (group: high/low contamination concern) × 6 (stimuli: CS-/GS1/GS2/GS3/GS4/CS+) repeated-measures ANOVA was performed. Then, we combined GS1/GS2, GS3/GS4 to perform a 2 (group: high/low contamination concern) × 3 (stimuli: GS1GS2/GS3GS4/CS+) repeated-measures ANOVA. Additionally, exploratory independent samples t-tests were conducted for each stimulus type (CS-/GS1/GS2/GS3/GS4/CS+) during the generalization phase to investigate differences in brain activation between the two groups in response to various stimuli.

#### ROI analysis

Based on previous research and the results of the second-order analysis in this study, region of interest (ROI) analysis focuses on exploring the differences in activation levels of the insula and putamen between the two groups (Gan et al., 2022; Gan et al., 2024; Wang, Sun, Becker, & Lei, 2022). The masks of insula (left and right) and putamen (left and right) were created using the AAL atlas (Tzourio-Mazoyer et al., 2002), implemented in WFU Pickatlas (Maldjian, Laurienti, Kraft, & Burdette, 2003). The mean percent signal changes within each ROI were extracted using the Marsbar toolbox (Brett et al., 2002) and subsequently exported to SPSS for analysis. The ROIs were then analyzed separately using a two-level group analysis, with group as a between-subjects variable and stimulus type as a within-subjects variable, employing a 2 (group: high/low contamination concern) × 6 (stimuli: CS-/GS1/GS2/GS3/GS4/CS+) repeated-measures ANOVA.

## Results

### Demographic results

Independent samples t-test was done for the age of subjects and the scores of each questionnaire in the two groups, and the statistical results and the gender comparison between the two groups are shown in Table 1. Among them, there is a significant difference between the two groups in the shares of PI-COWC, DSR and OCIR, indicating that the grouping is effective.

**Table 1.** Scores of PI-COWC, DSR, OCI-R, SAI, TAI and gender comparison between two groups

	HCC *M* ± *SD* (*N* = 27)	LCC *M* ± *SD* (*N* = 30)	Test statistic, sig.	
Age (year)	20.41 ± 1.45	19.80 ± 1.16	t = 1.76	p = 0.84
Gender (female %)	24 (88%)	23 (76%)	*χ^2^ =* 1.47	*p* = 0.23
PI-COWC	18.00 ± 3.38	4.23 ± 1.59	*t* = 19.30	*p* < 0.001***
DSR	66.44 ± 12.91	46.83 ± 13.78	*t* = 5.53	*p* < 0.001***
OCIR	35.59 ± 11.72	21.33 ± 9.56	*t* = 5.10	*p* < 0.001***
OCIR washing	9.78 ± 2.64	5.33 ± 1.77	*t* = 7.54	*p* < 0.001***
OCIR obsessing	9.33 ± 3.16	7.33 ± 2.55	*t* = 2.64	*p* = 0.01
OCIR hoarding	10.63 ± 2.57	9.43 ± 2.49	*t* = 1.78	*p* = 0.08
OCIR ordering	9.37 ± 2.54	7.11 ± 2.32	*t* = 3.52	*p* = 0.001
OCIR neutralizing	6.89 ± 3.42	5.03 ± 2.23	*t* = 2.45	*p* = 0.018
OCIR checking	9.26 ± 2.60	6.30 ± 2.29	*t* = 4.57	*p* < 0.001***
STAI-S	42.15 ± 12.61	34.77 ± 9.21	*t* = 2.54	*p* = 0.014*
STAI-T	45.44 ± 11.23	38.77 ± 10.35	*t* = 2.34	*p* = 0.023*

*Note:* M, mean; SD, standard deviation; N, number; STAI-T, Trait Anxiety Inventory; STAI-S, State Anxiety Inventory; PI-COWC, Padua Inventory-Contamination of obsessive-compulsive Disorder Subscale; DSR, The Disgust Scale-Revised; OCIR, The Obsessive–Compulsive Inventory-Revised; OCIR washing, The Washing Subscale of the Obsessive–Compulsive Inventory-Revised; OCIR obsessing, The Obsessing Subscale of the Obsessive–Compulsive Inventory-Revised; OCIR hoarding, The Hoarding Subscale of the Obsessive–Compulsive Inventory-Revised; OCIR ordering, The Ordering Subscale of the Obsessive–Compulsive Inventory-Revised; OCIR neutralizing, The Neutralizing Subscale of the Obsessive–Compulsive Inventory-Revised; OCIR checking, The Checking Subscale of the Obsessive–Compulsive Inventory-Revised; HCC, high contamination concern; LCC, low contamination concern.

*p < 0.05 **p < 0.01 ***p < 0.001

### Behavioral results

#### Acquisition

**Figure 2.**
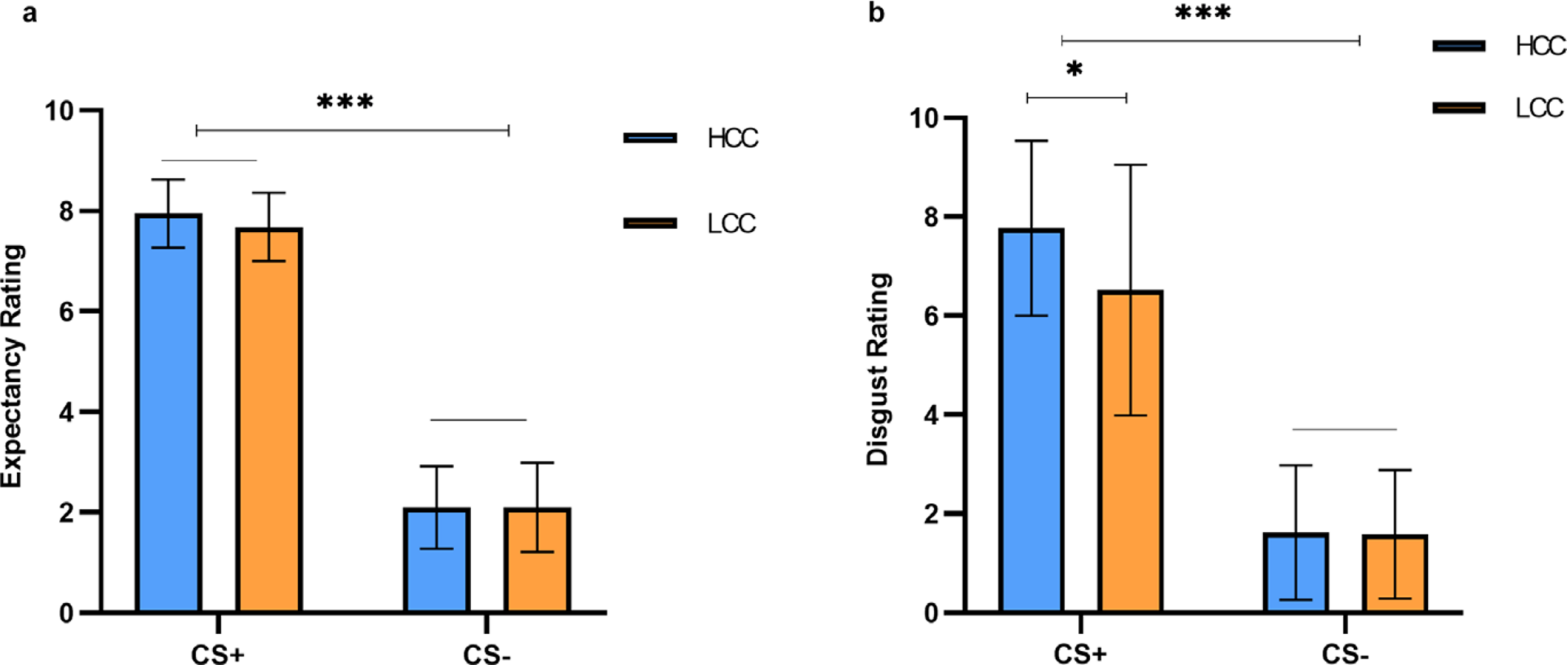
**Behavioral results during the acquisition stage**. (a) Acquisition phase rating scores for both groups of subjects. There were no between-group differences in the pattern of acquisition for both groups. (b) Disgust ratings to stimuli in both groups during the acquisition phase, subjects in the high compulsive tendency group had higher CS+ disgust ratings. Error bars reflect ± standard deviation (SD). ∗*p* < 0.05, ∗∗*p* < 0.01, ∗∗∗*p* < 0.001.

A 2 (group: high/low contamination concern) × 2 (stimulus: CS+/CS-) ANOVA on the expected ratings of the two groups during the acquisition phase revealed a significant main effect of stimulus type [*F* (1,55) = 976.70, *p* < 0.001, *η_p_^2^* = 0.947]. Post hoc analyses indicated that participants rated CS+ (*M* ± *SD* = 7.82 ± 0.09) significantly higher than CS− (*M* ± *SD* = 2.10 ± 0.11), demonstrating successful acquisition of the experimental rule (see Figure 2a). However, the differences between groups were not significant, nor was the interaction between group and stimulus type.

In a 2 (group: high/low contamination concern) × 2 (stimulus: CS+/CS−) repeated-measures ANOVA on the disgust ratings of the different CSs during the acquisition phase (The data of the two individuals was not recorded), a significant main effect of stimulus type was observed [*F* (1,53) = 288.70, *p* < 0.001, *η_p_^2^* = 0.845]. Further analysis revealed that CS+ was rated significantly higher in disgust (*M* ± *SD* = 7.14 ± 0.29) compared to CS− (*M* ± *SD* = 1.60 ± 0.18). A borderline significant interaction between group and stimulus type was also detected [*F* (1,53) = 3.51, *p* = 0.066, *η_p_^2^* = 0.062]. Simple effects analysis showed that high-compulsive tendency participants rated CS+ significantly higher in disgust (*M* ± *SD* = 7.77 ± 0.42) compared to low-compulsive tendency participants (*M* ± *SD* = 6.52 ± 0.40; *p* = 0.035). The main effect of the group was not significant (see Figure 2b).

#### Generalization

**Figure 3.**
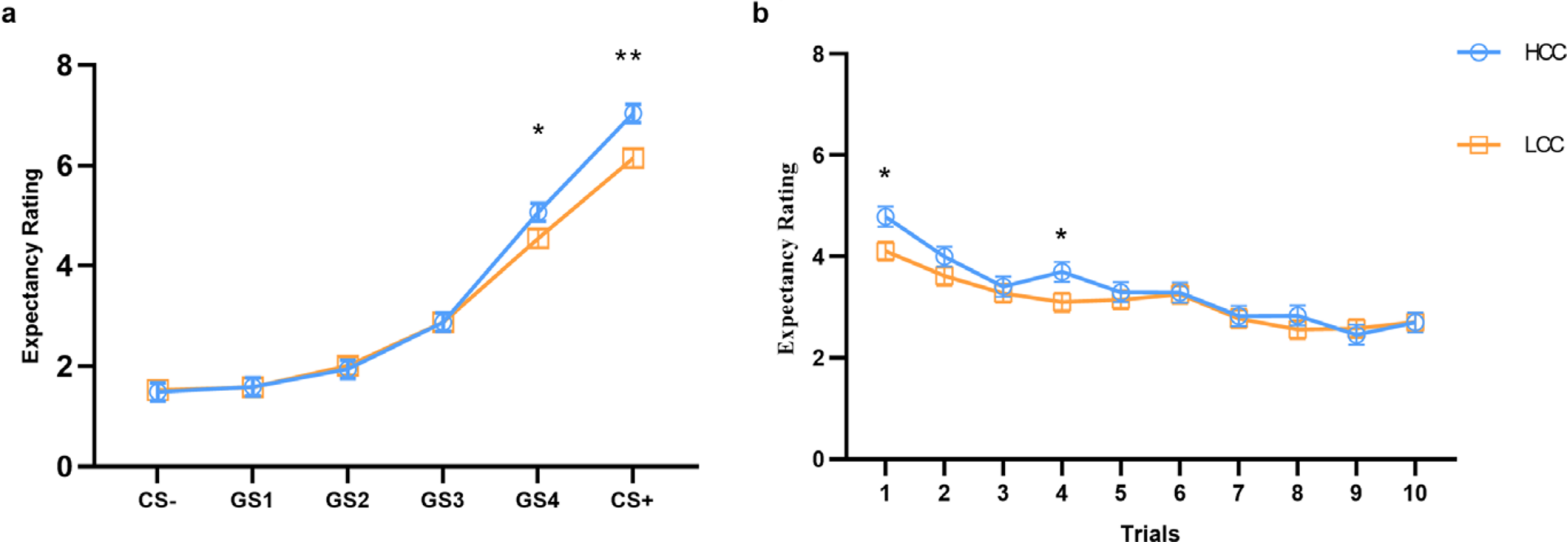
**Subjects’ expectancy ratings of different types of stimuli during the generalization phase.** (a) Shows the ratings of the two groups of subjects for different stimulus types. (b) Demonstrates the difference in overall scores between the two groups of subjects across trials. Error bars reflect ± Standard deviation (SD).

The results of the linear mixed model analysis revealed a nonsignificant interaction among group, stimulus type and trial. However, there was a significant interaction between group and stimulus type [*χ^2^* (54) = 165, *p* < 0.001, *η_p_^2^* = 0.01]. Simple effects analysis showed that participants in the high obsessive–compulsive tendency group rated CS+ significantly higher than low obsessive–compulsive tendency group [*t* (94.4) = 3.52, *p* = 0.007]. A similar pattern was observed for GS4, the stimulus most similar to CS+ [*t* (94.4) = 2.05, *p* = 0.0429], indicating that individuals with high contamination concern are more sensitive to disgusting stimuli. Additionally, the group × trial interaction was significant [*χ^2^* (50) = 187.5, *p* < 0.001, *η_p_^2^* = 0.006]. Simple effects analysis revealed that the high obsessive–compulsive tendency group had significantly higher ratings in the early phase, specifically during the first trial [*t* (133) = 2.47, *p* = 0.0147] and the fourth trial [*t* (133) = 2.20, *p* = 0.03], see Figure 3.

In a 2 (group: high/low contamination concern) × 6 (stimulus: CS+/CS-/GS1/GS2/GS3/GS4) repeated-measures ANOVA on the disgust ratings of the different stimuli during the generalization phase (The data of the one individual was not recorded), a significant main effect of stimulus type was observed [*F* (5,270) = 143.07, *p* < 0.001, *η_p_^2^* = 0.726]. Post hoc analyses revealed that CS+ was rated highest in disgust (*M* ± *SD* = 6.94 ± 0.30), and the ratings from high to low are GS4 (*M* ± *SD* = 4.68 ± 0.32), GS3 (*M* ± *SD* = 2.62 ± 0.25), GS2 (*M* ± *SD* = 1.80 ± 0.17), GS1 (*M* ± *SD* = 1.34 ± 0.10) and CS− (*M* ± *SD* = 1.30 ± 0.10). The main effect of the group was not significant, and there was no significant interaction.

### fMRI results

#### Results of whole-brain analysis of the generalization phase

The interaction between group and stimulus type was entered into the flexible factorial design module to examine the main effect of stimulus type. Whole-brain analysis revealed no significant group-by-stimulus interaction. See more information in supplementary materials.

To further investigate differences in brain activation during the disgust generalization phase between individuals with high and low contamination concerns when exposed to various stimuli, independent samples t-tests were conducted for each GS. The results indicated that the high contamination concern group exhibited significantly higher engagement of the right putamen and left anterior insula (FWE-corrected, *p* < 0.05) under the GS4 condition, which is the most similar to CS+. The activated brain regions are detailed in Table 2.

**Table 2.** Brain regions by Independent sample t-test under GS4 conditions

Brain region (High>Low)	Cluster level			MNI coordinates			T-value
	PFWE-corr	kE	puncorr	X	Y	Z	
R.Putamen	0.010	87	0.001	24	9	6	5.26
L‥AIns anterior insula	0.000	175	0.000	−36	3	9	4.98

#### ROI results

A significant main effect of stimulus type was observed in the left insula [*F* (5,275) = 13.80, *p* < 0.001, *η_p_^2^* = 0.201]. Post hoc analyses revealed that both groups exhibited the highest level of activation in this region when exposed to CS+ (*M* ± *SD* = 0.53 ± 0.05). Additionally, the interaction between the group and stimulus type was significant [*F* (5,275) = 2.70, *p* = 0.021, *η_p_^2^* = 0.047]. Simple effects analyses indicated that the left insula activation was significantly greater in the high-contamination concern group than in the low-contamination concern group when exposed to GS4 (*p* = 0.018). There is no significant difference between groups.

A significant main effect of stimulus type was observed in the left putamen region [*F* (4.26,233.92) = 3.19, *p* = 0.012, *η_p_^2^* = 0.055]. Simple effects analysis revealed that both groups exhibited the highest level of activation in this region when exposed to CS+ (*M* ± *SD* = 0.32 ± 0.037). The main effect of the group was also significant [*F* (1,55) = 7.78, *p* = 0.007, *η_p_^2^* = 0.124], with post hoc analyses indicating that the high contamination concern group (*M* ± *SD* = 0.34 ± 0.04) had significantly greater activation levels in the left putamen compared to the low contamination concern group (*M* ± *SD* = 0.17 ± 0.042). Furthermore, a significant group × stimulus type interaction was found [*F* (4.25,233.92) = 3.07, *p* = 0.015, *η_p_^2^* = 0.053]. Simple effects analyses showed significantly greater activation in the left putamen in the high contamination concern group compared to the low contamination concern group when exposed to GS4 (*p* < 0.001) and GS3 (*p* = 0.023) and CS+(*p* = 0.021).

**Figure 4.**
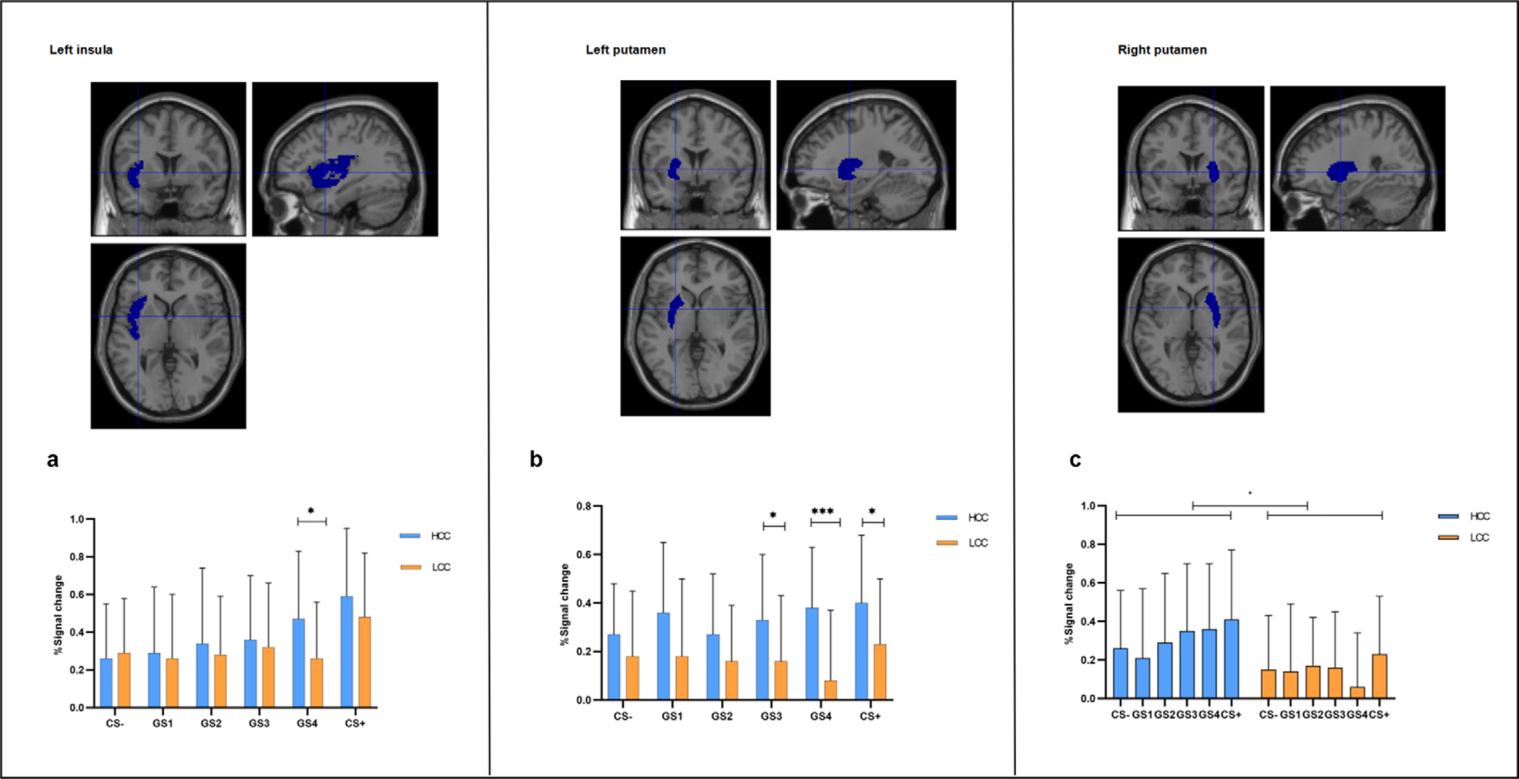
**Activation of the left insula and Bilateral putamen.** (a) Activation of left insula between two groups. (b) Activation of left putamen between two groups. (c) Activation of right putamen between two groups. Error bars reflect ± Standard deviation (SD). *p* < 0.05, *p* < 0.01, *p* < 0.001.

In addition, significant differences were observed in the activation levels of the right putamen between the two groups [*F* (1,55) = 5.77, *p* = 0.02, *η_p_^2^* = 0.095]. Post hoc analyses indicate that the high contamination concern group (*M* ± *SD* = 0.31 ± 0.054) had significantly greater activation levels in the left putamen compared to the low contamination concern group (*M* ± *SD* = 0.15 ± 0.051), see Figure 4.

#### Correlation between behavioral and brain responses

Based on the above results, we conducted a correlation analysis on the entire sample to investigate the correlation between brain activation, scale scores and behavioral data (Xu et al., 2020; Xu et al., 2021). The results showed that the activation of left insula and putamen were positively correlated with the scale scores of the subjects and also positively correlated with the expectancy rating of CS+ during the generalization stage (see more details in supplementary material).

## Discussion

The present study investigated the cognitive neural mechanisms underlying disgust generalization in individuals with high and low compulsive contamination concerns. Results from the acquisition phase indicated that both groups successfully learned disgusting associations, with no significant between-group differences in US expectancy, consistent with previous findings (Armstrong & Olatunji, 2017). However, disgusting scores were significantly higher in the high-contamination concern group compared to the low-contamination concern group. During the generalization phase, behavioral data revealed that individuals with high contamination concern exhibited higher anticipatory ratings for disgusting stimuli, particularly for CS+ and GS4. Furthermore, fMRI data indicated that individuals with high contamination concern showed significantly greater activation in the left insula when exposed to GS4, as well as increased activation in the putamen. These activation reactions are correlated with scale scores and behavioral data.

During the acquisition phase, no significant difference was observed between the two groups in terms of anticipation scores. However, a notable difference emerged in disgust scores, with the high contamination concern group exhibiting significantly greater disgust compared to the low contamination concern group. This suggests that individuals with high contamination concern exhibit a stronger response to negative stimuli, aligning with their heightened sensitivity to such stimuli, as documented in prior studies (Olatunji et al., 2011; Olatunji et al., 2017). In the generalization phase, the high contamination concern group reported higher expectancy ratings for the CS+ and GS4. This finding supports the notion that one of the core cognitive dysfunctions in OCD is an exaggerated perception of threat (Olatunji et al., 2007). The tendency of individuals with OCD to overreact to stimuli may be attributed to the generalization of responses from threat cues to neutral or safe stimuli. The findings demonstrate heightened reactivity in individuals with high compulsive contamination concerns during the generalization phase, providing further evidence for this theory.

One potential explanation for this phenomenon is that individuals with high contamination concerns are more sensitive to stimuli that resemble disgusting cues, leading them to respond similarly to the generalized stimuli in a proactive and self-protective manner (Struyf, Zaman, Vervliet, & Van Diest, 2015). Alternatively, the similarity of GS4 to the CS+ may heighten attention and vigilance in individuals with high contamination concerns, resulting in elevated appraisal and awareness. The two-phase model suggests that disgusting stimuli first capture attention, but are then increasingly avoided, potentially leading to heightened sensitivity to stimuli similar to the CS+ (Fink-Lamotte, Svensson, Schmitz, & Exner, 2022; Knowles, Cox, Armstrong, & Olatunji, 2019).

Regarding brain activation, the ROI analysis revealed that individuals with high-contamination concerns exhibited increased activation in the left insula during the generalization phase when presented with GS4. The insula is a critical brain region for disgust learning, playing a central role in interoception and the processing of disgust (Berlin et al., 2015; Song et al., 2011). Additionally, studies have shown that serotonin (5-HT) within the insula is involved in regulating anticipatory disgust, a critical component of disgust learning (Limebeer, Rock, Sharkey, & Parker, 2018; Tuerke, Limebeer, Fletcher, & Parker, 2012).

Furthermore, research has indicated that the insula exhibits greater activation intensity during disgust learning compared to fear learning, highlighting its distinct role in conditioned learning processes (Klucken et al., 2012; Klucken et al., 2013). The findings of this study extend this understanding by demonstrating the insula’s role in disgust generalization, particularly in individuals with high contamination concern, thereby providing insights into the neural mechanisms underlying this phenomenon.

In addition to the insula, ROI analyses revealed that the bilateral putamen exhibited higher levels of activation in individuals with high contamination concerns. Numerous studies have demonstrated that viewing disgusting images increases neural activity in the basal ganglia, particularly the caudate nucleus and putamen (Gan et al., 2022; Gan et al., 2024). This activation also intensifies when subjects recall or re-experience the disgusting event, suggesting that the basal ganglia, including the putamen, play a crucial role in the processing of disgusting experiences (Fitzgerald et al., 2004), and the circuitry connecting the basal ganglia nodes with prefrontal regions are critically involved in inhibitory control (Zhuang et al., 2021; Zhuang et al., 2023). Recent research has further indicated that enhanced activation of the putamen is associated with an individual’s ability to recognize disgust-inducing faces, highlighting the importance of putamen in processing disgust-related emotions (Labuschagne et al., 2018). These findings align with this study’s observation that the high-contamination concern group exhibited a stronger response to disgust stimuli and activation in the putamen. Consequently, these results together emphasize the importance of the putamen and insula disgust processing in general as well as in pathological alterations of disgust.

Our findings suggest that individuals with high contamination concerns exhibit greater disgust generalization and heightened neural activity in regions such as the insula and putamen. This heightened excessive generalization may contribute to the difficulty of treating disgust in therapeutic settings. According to the “law of contagion,” when an object comes into contact with something perceived as contaminated, it is believed to retain that contamination indefinitely. For example, if a clean spoon falls onto a floor considered dirty, it is seen as permanently contaminated, regardless of any cleaning attempts afterwards (Rozin, Millman, & Nemeroff, 1986). Since individuals with OCD tend to excessively generalize disgust to GS, incorporating these GS alongside the original CS in exposure therapy may enhance therapeutic outcomes. Gradually introducing both the CS and similar GS in controlled exposure sessions can help patients reduce their overgeneralized disgust responses and better manage their reactions to a wider range of stimuli, potentially improving the effectiveness of treatment for contamination-related fears.

However, this study still has some limitations. On the one hand, besides brain activation data, this study did not measure other physiological indicators such as heart rate and sympathetic skin response. Future research can explore multiple physiological indicators in combination. On the other hand, the participants in this study were drawn from a nonclinical population; therefore, further research is required to determine whether these findings can be generalized to clinical populations.

In summary, this study investigated the characteristics and neural mechanisms of disgust generalization in individuals with high and low contamination concerns. We found that individuals with high contamination concerns exhibited greater generalization and increased activation in the insula and putamen in response to GS4, which closely resembles CS+. These results enhance our understanding of the mechanisms underlying disgust generalization in individuals with contamination concern and provide a foundation for further exploration of obsessive–compulsive symptoms.

## Supporting information

Liu et al. supplementary material Liu et al. supplementary material

## Data Availability

All data are available upon reasonable request.

## Supplementary material

The supplementary material for this article can be found at http://doi.org/10.1017/S0033291725000728.
