# Enhanced disgust generalization in obsessive–compulsive disorder is related to insula and putamen hyperactivity – CORRIGENDUM

**DOI:** 10.1017/S0033291726105285

**Published:** 2026-07-13

**Authors:** Juntong Liu, Jinxia Wang, Yuchen Song, Benjamin Becker, Xianchao Ming, Yi Lei

This article was published with an incorrect funding number.

In the published version, the funding number was listed as STI 2030—Major Projects [2022ZD021090]. The correct funding number should be STI 2030—Major Projects [2022ZD0210900].

The authors apologise for this error.
