# Supplementary material for: Enhanced disgust generalization in obsessive–compulsive disorder is related to insula and putamen hyperactivity
Source: Psychol Med. 2025 Apr 14;55:e116. doi: 10.1017/S0033291725000728 (PMC12094653; doi:10.1017/S0033291725000728)
Supplement: Liu et al. supplementary material [file S0033291725000728sup001.docx]

**Supplementary** **materials**

**Method and Materials**

**Supplementary materials of experimental materials**

According to previous research, contamination OCD is closely related to pathogen disgust (Olatunji et al., 2015). Therefore, we selected 12 different images directly related to pathogenic aversion as US stimuli, including death, animals, food, hygiene, body products, and envelope violations (Wang et al., 2024). These images were selected from a previously established library of disgusting images and further selected by Gan et al(2024) (Gan et al., 2024; Haberkamp et al., 2017).

**Supplementary materials of fMRI data acquisition**

Before entering the MRI chamber, subjects were asked to change into laboratory-specific clothing to avoid metal objects that could affect their safety and the quality of the imaging. They were also instructed to remove any metal jewelry, hairpins, or dentures. Special earplugs were provided to reduce noise interference during the operation of the equipment, and foam blocks were used to minimize head movements. Subjects were instructed to keep their heads still and avoid crossing their arms and legs during the entire scanning process.

The fMRI data for this experiment were acquired using a Siemens Prisma 3.0T MRI scanner (Siemens, Erlangen, Germany). The experimental materials and procedures were presented via E-prime 2.0, and subjects viewed the experimental procedures through a projector. High-resolution T1-weighted structural images and one sequence of functional images were acquired for each subject.

The BOLD functional image sequence was performed using an echo-planar imaging (EPI) sequence with a repetition time (TR) of 2000 ms and an echo time (TE) of 30 ms. The flip angle was set to 90°, with a field of view (FOV) of 224 mm × 224 mm, a resolution matrix of 64 × 64, voxel sizes of 2 × 2 × 2 mm³, and a slice thickness of 2 mm, with 62 axial slices.

High-resolution T1-weighted images were obtained using a fast echo sequence (MP-RAGE) with a TR of 2530 ms, a TE of 2.98 ms, a flip angle of 7°, 192 axial slices, an FOV of 256 mm × 256 mm, a resolution matrix of 64 × 64, voxel sizes of 0.5 × 0.5 × 1 mm³, and a slice thickness of 1.0 mm.

**Supplementary materials of fMRI data preprocessing**

The pre-processing steps included:

Conversion: Transforming T1 structural images and task-state functional images from DICOM format to NIFTI format.

Slice Timing Correction: Parameters were set as follows: Total Number of Slices = 62, Repetition Time (TR) = 2 s, Time Acquisition (TA) = TR - (TR/62) = 1.967 s, and Slice Order: [2, 33, 4, 35, 6, 37, 8, 39, 10, 41, 12, 43, 14, 45, 16, 47, 18, 49, 20, 51, 22, 53, 24, 55, 26, 57, 28, 59, 30, 61, 1, 32, 3, 34, 5, 36, 7, 38, 9, 40, 11, 42, 13, 44, 15, 46, 17, 48, 19, 50, 21, 52, 23, 54, 25, 56, 27, 58, 29, 60, 31, 62], with the reference slice set to the intermediate layer (32).

Head Movement Correction: The average head image was used as a reference. Rejection criteria were set to a maximum head translation of ±3 mm and a maximum head rotation of ±3°. One subject was excluded based on these criteria.

Spatial Segmentation: Each subject's T1 structural image was used to segment gray matter, white matter, and cerebrospinal fluid in the functional images.

Spatial Alignment: Each subject’s T1 structural image was aligned to the MNI standard space, with spatial dimensions set to Bounding Box = [-90, -126, -72; 90, 90, 108] and voxel sizes of 3 × 3 × 3 mm³.

Spatial Smoothing: Gaussian smoothing was applied with a kernel size of 6 × 6 × 6 mm³. After smoothing, alignment was performed again with the Bounding Box unchanged and voxel sizes of 1 × 1 × 1 mm³. The preprocessed fMRI images were saved in NIFTI format with the prefix "wuaf".

**Reference**

Burns, G. L., Keortge, S. G., Formea, G. M., & Sternberger, L. G. (1996). Revision of the Padua Inventory of obsessive compulsive disorder symptoms: distinctions between worry, obsessions, and compulsions. *Behav Res Ther*, *34*(2), 163-173. <https://doi.org/10.1016/0005-7967(95)00035-6>

Foa, E. B., Huppert, J. D., Leiberg, S., Langner, R., Kichic, R., Hajcak, G., & Salkovskis, P. M. (2002). The Obsessive-Compulsive Inventory: development and validation of a short version. *Psychol Assess*, *14*(4), 485-496.

Gan, X., Zhou, F., Xu, T., Liu, X., Zhang, R., Zheng, Z., . . . Becker, B. (2024). A neurofunctional signature of subjective disgust generalizes to oral distaste and socio-moral contexts. *Nat Hum Behav*, *8*(7), 1383-1402. <https://doi.org/10.1038/s41562-024-01868-x>

Haberkamp, A., Glombiewski, J. A., Schmidt, F., & Barke, A. (2017). The DIsgust-RelaTed-Images (DIRTI) database: Validation of a novel standardized set of disgust pictures. *Behav Res Ther*, *89*, 86-94. <https://doi.org/10.1016/j.brat.2016.11.010>

Olatunji, B. O., Ebesutani, C., & Kim, E. H. (2015). Examination of a bifactor model of the Three Domains of Disgust Scale: specificity in relation to obsessive-compulsive symptoms. *Psychol Assess*, *27*(1), 102-113. <https://doi.org/10.1037/pas0000039>

Olatunji, B. O., Williams, N. L., Tolin, D. F., Abramowitz, J. S., Sawchuk, C. N., Lohr, J. M., & Elwood, L. S. (2007). The Disgust Scale: item analysis, factor structure, and suggestions for refinement. *Psychol Assess*, *19*(3), 281-297. <https://doi.org/10.1037/1040-3590.19.3.281>

Sydeman, S. (2018). State-Trait Anxiety Inventory. In V. Zeigler-Hill & T. K. Shackelford (Eds.), *Encyclopedia of Personality and Individual Differences* (pp. 1-3). Springer International Publishing. <https://doi.org/10.1007/978-3-319-28099-8_950-1>

Wang, J., Becker, B., Wang, Y., Ming, X., Lei, Y., & Wikgren, J. (2024). Conceptual-level disgust conditioning in contamination-based obsessive-compulsive disorder. *Psychophysiology*, e14637. <https://doi.org/10.1111/psyp.14637>

**Figures**


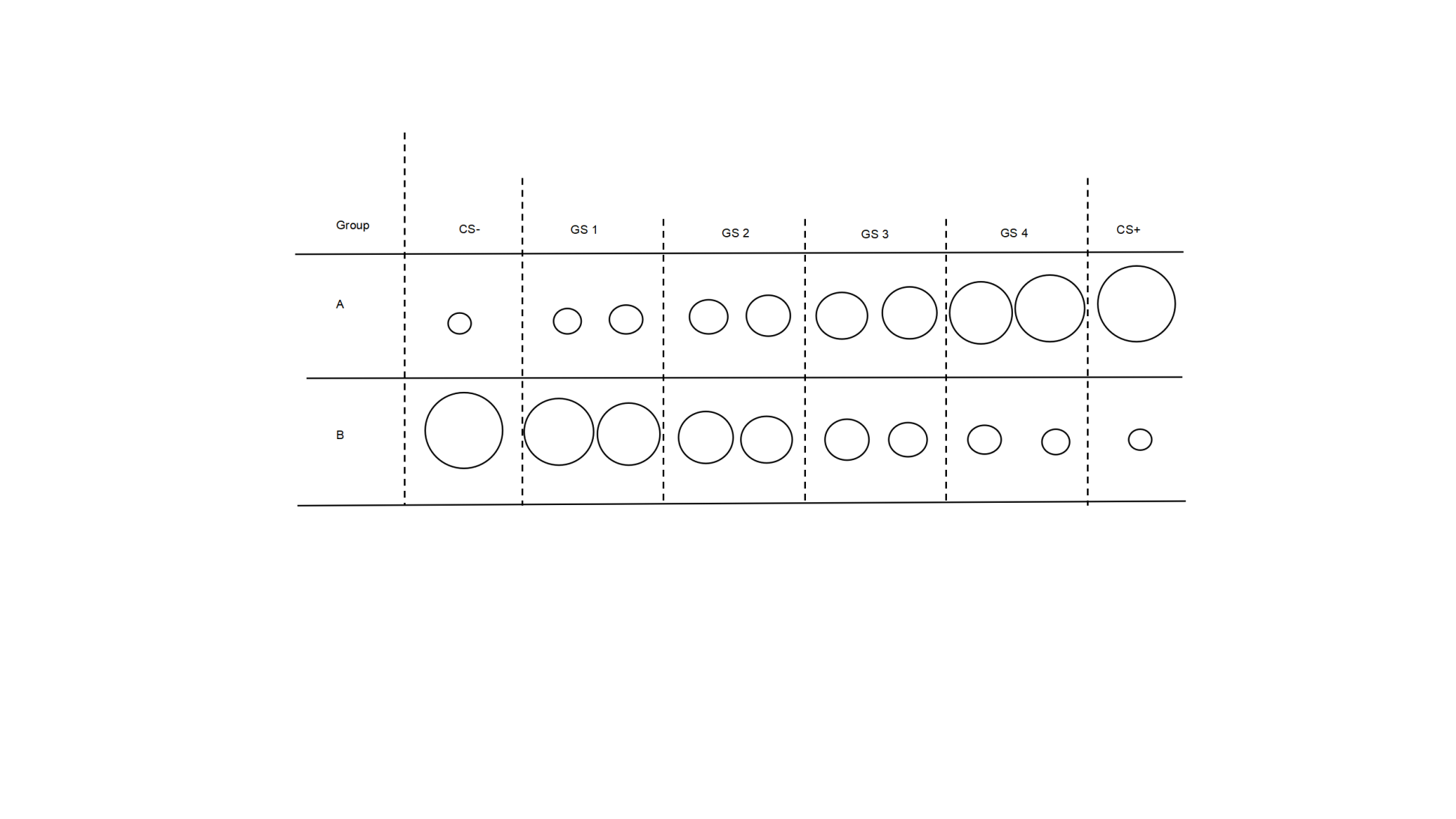


Fig. S1. Examples of conditioned and generalized stimulus materials(CS & GS).


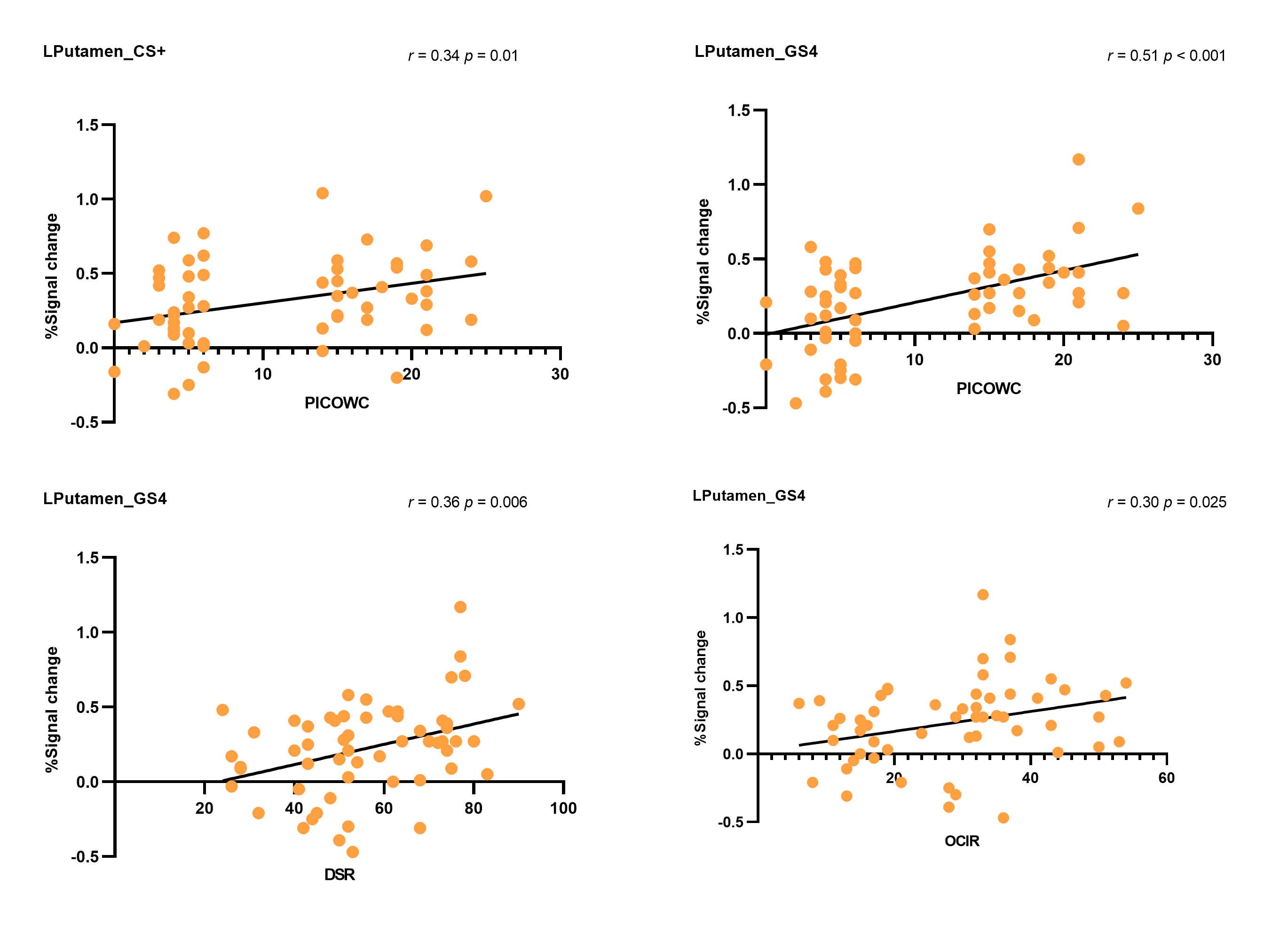


Fig. S2. **Correlation between PICOWC, DSR & OCIR scores and brain activities in left putamen.** Brain activities are reflected by mean percentage signal changes of each condition relative to the baseline. RPutamen_GS4 represents brain activities of right putamen in GS4 conditions during generalization stage. RPutamen_CS+ represents brain activities of right putamen in CS+ conditions during generalization stage. LPutamen_GS4 represents brain activities of left putamen in GS4 conditions during generalization stage.


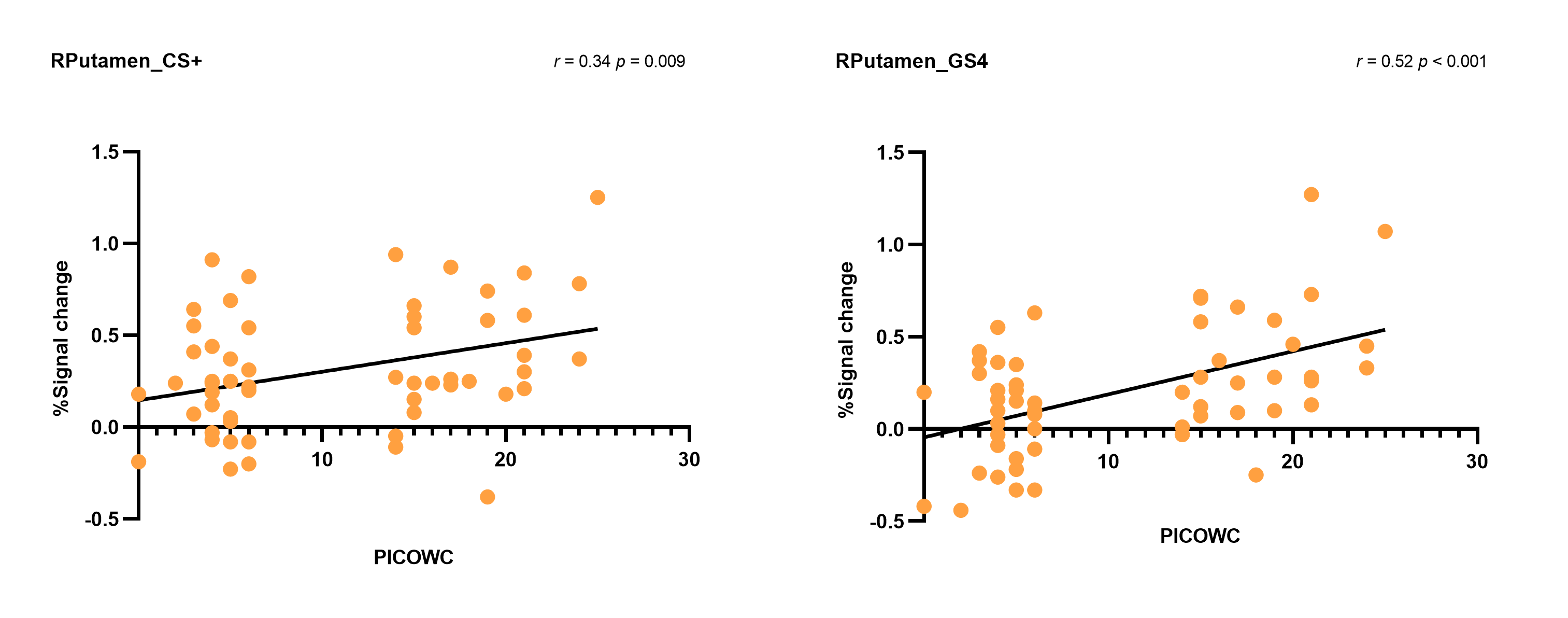


Fig. S3. **Correlation between PICOWC scores and brain activities in right putamen**. Brain activities are reflected by mean percentage signal changes of each condition relative to the baseline. RPutamen_GS4 represents brain activities of right putamen in GS4 conditions during generalization stage. RPutamen_CS+ represents brain activities of right putamen in CS+ conditions during generalization stage.


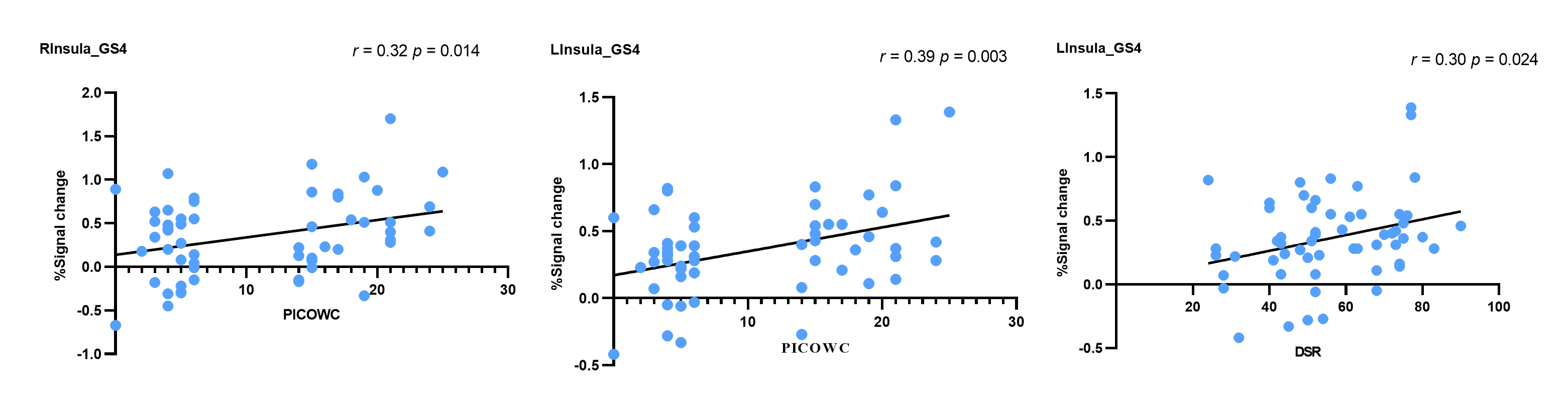


Fig. S5. **Correlation between PICOWC & DSR scores and brain activities in insula.** Brain activities are reflected by mean percentage signal changes of each condition relative to the baseline. RInsula_GS4 represents brain activities of right insula in GS4 conditions during generalization stage. LInsula_GS4 represents brain activities of left insula in GS4 conditions during generalization stage.

**Supplementary materials of whole-brain analysis of the generalization phase**

Stimulus type significantly activated certain brain regions after applying FWE correction, as detailed in Supplementary Table S2 below.

| Table S1. Brain regions by the main effect of stimulus types | | | | | | | |
| --- | --- | --- | --- | --- | --- | --- | --- |
| Brain region | Cluster-level | | | MNI coordinates | | | F-value |
|  | PFWE-corr | kE | puncorr | X | Y | Z |  |
| R.AIns anterior insula | 0.000 | 20701 | 0.000 | 30 | 24 | 0 | 45.42 |
| L.Cerebellum Exterior | 0.001 | 102 | 0.000 | -30 | -72 | -54 | 15.2 |
| Cerebellar vermal Lobules | 0.000 | 151 | 0.000 | -3 | -57 | -39 | 12.00 |
| Superior parietal lobule | 0.000 | 331 | 0.000 | 18 | -48 | 69 | 10.02 |
| L.superior temporal gyrus | 0.000 | 221 | 0.000 | -63 | -15 | 3 | 7.63 |
| L.Hippocampus | 0.049 | 44 | 0.004 | -24 | -15 | -21 | 6.42 |

| Table S2. Brain regions by the main effect of stimulus types | | | | | | | |
| --- | --- | --- | --- | --- | --- | --- | --- |
| Brain region | Cluster-level | | | MNI coordinates | | | F-value |
|  | PFWE-corr | kE | puncorr | X | Y | Z |  |
| R.AIns anterior insula | 0.000 | 20701 | 0.000 | 30 | 24 | 0 | 45.42 |
| L.Cerebellum Exterior | 0.001 | 102 | 0.000 | -30 | -72 | -54 | 15.2 |
| Cerebellar vermal Lobules | 0.000 | 151 | 0.000 | -3 | -57 | -39 | 12.00 |
| Superior parietal lobule | 0.000 | 331 | 0.000 | 18 | -48 | 69 | 10.02 |
| L.superior temporal gyrus | 0.000 | 221 | 0.000 | -63 | -15 | 3 | 7.63 |
| L.Hippocampus | 0.049 | 44 | 0.004 | -24 | -15 | -21 | 6.42 |

| Table S3-1. Correlations among scale scores, behavioral data and brain activation in left putamen. | | | | | | | | | | | |
| --- | --- | --- | --- | --- | --- | --- | --- | --- | --- | --- | --- |
|  |  | PICOW-C | DSR | OCIR | G_CS+ | Left-putamen | | | | | |
|  |  |  |  |  |  | CS+ | CS- | GS1 | GS2 | GS3 | GS4 |
|  | PICOW-C | 1 |  |  |  |  |  |  |  |  |  |
|  | DSR | 0.67** | 1 |  |  |  |  |  |  |  |  |
|  | OCIR | 0.62** | 0.57** | 1 |  |  |  |  |  |  |  |
|  | G_CS+ | 0.29* | 0.25 | 0.12 | 1 |  |  |  |  |  |  |
| Left-putamen | CS+ | .34** | 0.21 | 0.27* | 0.15 | 1 |  |  |  |  |  |
|  | CS- | 0.27* | 0.27* | 0.14 | 0.26 | 0.72** | 1 |  |  |  |  |
|  | GS1 | 0.32* | 0.30* | 0.20 | 0.34** | 0.66** | 0.66** | 1 |  |  |  |
|  | GS2 | 0.29* | 0.18 | 0.22 | 0.22 | 0.63** | 0.77** | 0.71** | 1 |  |  |
|  | GS3 | 0.38** | 0.30* | 0.18 | 0.30* | 0.66** | 0.76** | 0.75** | 0.75** | 1 |  |
|  | GS4 | 0.51** | 0.36** | 0.30* | 0.22 | 0.60** | 0.69** | 0.67** | 0.68** | 0.80** | 1 |

Note. PICOWC = Padua Inventory-Contamination of obsessive compulsive Disorder Subscale; DSR = The Disgust Sensitivity Scale; OCIR = The Revised obsessive compulsive Inventory; G_CS+ = Behavioral data of CS+ in the generalization stage.

| Table S3-2. Correlations among scale scores, behavioral data and brain activation in right putamen. | | | | | | | | | | | |
| --- | --- | --- | --- | --- | --- | --- | --- | --- | --- | --- | --- |
|  |  | PICOW-C | DSR | OCIR | G_CS+ | Right-putamen | | | | | |
|  |  |  |  |  |  | CS+ | CS- | GS1 | GS2 | GS3 | GS4 |
|  | PICOW-C | 1 |  |  |  |  |  |  |  |  |  |
|  | DSR | 0.67** | 1 |  |  |  |  |  |  |  |  |
|  | OCIR | 0.62** | 0.57** | 1 |  |  |  |  |  |  |  |
|  | G_CS+ | 0.29* | 0.25 | 0.12 | 1 |  |  |  |  |  |  |
| Right-putamen | CS+ | .34* | 0.20 | 0.24 | 0.21 | 1 |  |  |  |  |  |
|  | CS- | 0.29* | 0.24 | 0.12 | 0.28* | 0.71** | 1 |  |  |  |  |
|  | GS1 | 0.28* | 0.22 | 0.17 | 0.32** | 0.67** | 0.67** | 1 |  |  |  |
|  | GS2 | 0.28* | 0.16 | 0.17 | 0.28* | 0.69** | 0.82** | 0.77** | 1 |  |  |
|  | GS3 | 0.39** | 0.24 | 0.22 | 0.34** | 0.73** | 0.76** | 0.79** | 0.76** | 1 |  |
|  | GS4 | 0.50** | 0.28* | 0.27* | 0.30* | 0.66** | 0.78** | 0.73** | 0.73** | 0.82** | 1 |

Note. PICOWC = Padua Inventory-Contamination of obsessive compulsive Disorder Subscale; DSR = The Disgust Sensitivity Scale; OCIR = The Revised obsessive compulsive Inventory; G_CS+ = Behavioral data of CS+ in the generalization stage.
